# Sociodemographic disparities in concomitant left atrial appendage occlusion during cardiac valve operations

**DOI:** 10.1371/journal.pone.0286337

**Published:** 2023-05-25

**Authors:** Ayesha P. Ng, Nikhil Chervu, Yas Sanaiha, Amulya Vadlakonda, Elsa Kronen, Peyman Benharash

**Affiliations:** 1 Cardiovascular Outcomes Research Laboratories, David Geffen School of Medicine at UCLA, Los Angeles, CA, United States of America; 2 Department of Surgery, David Geffen School of Medicine at UCLA, Los Angeles, CA, United States of America; 3 Division of Cardiac Surgery, Department of Surgery, David Geffen School of Medicine at UCLA, Los Angeles, CA, United States of America; Hillel Yaffe Medical Center, ISRAEL

## Abstract

**Background:**

Sociodemographic disparities in atrial fibrillation (AF) management and thromboembolic prophylaxis have previously been reported, which may involve inequitable access to left atrial appendage occlusion (LAAO) during cardiac surgery. The present study aimed to evaluate the association of LAAO utilization with sex, race, and hospital region among patients with AF undergoing heart valve operations.

**Methods:**

Adults with AF undergoing valve replacement/repair in the 2012–2019 National Inpatient Sample were identified and stratified based on concurrent LAAO. Multivariable linear and logistic regressions were developed to identify factors associated with LAAO utilization. Mortality, complications including stroke and thromboembolism, hospitalization costs and length of stay (LOS) were secondarily assessed.

**Results:**

Of 382,580 patients undergoing valve operations, 18.7% underwent concomitant LAAO. Over the study period, the proportion of female patients receiving LAAO significantly decreased from 44.8% to 38.9% (p<0.001). Upon risk adjustment, female (AOR 0.93 [95% CI 0.89–0.97]) and Black patients (0.91 [0.83–0.99]) had significantly reduced odds of undergoing LAAO compared to males and Whites, respectively. Additionally, hospitals in the Midwest (1.38 [1.24–1.51]) and West (1.26 [1.15–1.36]) had increased likelihood of LAAO whereas Northeast hospitals (0.85 [0.77–0.94)] had decreased odds relative to the South. Furthermore, LAAO was associated with decreased stroke (0.71 [0.60–0.84]) and thromboembolism (0.68 [0.54–0.86]), $4,200 reduction in costs and 1-day decrement in LOS.

**Conclusions:**

Female and Black patients had significantly lower odds while Midwest and Western hospitals had greater odds of LAAO utilization. Enhancing access to LAAO during valvular surgery is warranted to improve clinical and financial outcomes for patients with AF.

## Introduction

Atrial fibrillation (AF), the most common sustained arrhythmia, is associated with a 5-fold increase in risk of thromboembolic stroke [1, 2]. Although long-term anticoagulation therapy is the mainstay for stroke prevention, as few as half of all eligible patients use anticoagulation due to contraindications including history of bleeding and concerns with management and quality of life [3, 4]. In nonvalvular AF, over 90% of strokes originate from the left atrial appendage, and its occlusion (LAAO) has been frequently proposed as an alternative to anticoagulation [5]. While LAAO is primarily recommended at the time of mitral valve and antiarrhythmic operations, its use is known to vary widely among physicians [6, 7].

Important sociodemographic disparities in AF management and outcomes of LAAO have been previously described. Black patients have been shown to be underrepresented among patients receiving percutaneous LAAO and have increased complications compared to Whites [8]. In addition, female patients undergoing LAAO with the Watchman device experienced higher risk of postoperative bleeding and mortality compared to males [9]. Geographic variation also exists and higher AF-related stroke mortality rates have been reported in the South compared to other regions of the United States [10]. However, disparities in LAAO utilization in the context of concomitant valvular heart surgery have yet to be examined. Given the growing evidence supporting thromboembolic prophylaxis with LAAO during cardiac surgery, it is necessary to characterize patterns of access to LAAO in order to understand potential inequities in outcomes [11].

The present study used a nationally representative database to evaluate the association of LAAO utilization with sex, race, and hospital region among patients with AF undergoing heart valve operations. In addition, we examined the influence of LAAO utilization on in-hospital clinical and financial outcomes. We hypothesized that female, Black, and Southern patients would be associated with reduced utilization of LAAO and increased in-hospital mortality, complications, length of stay, and hospitalization costs.

## Methods

This was a cross-sectional study using data from the 2012–2019 National Inpatient Sample (NIS). Maintained by the Healthcare Cost and Utilization Project (HCUP), the NIS is the largest publicly available all-payer inpatient database in the United States (US) and samples 20% of all hospital discharges [12]. Using robust survey-weighting algorithms, the NIS provides accurate estimates for approximately 97% of all hospitalizations in the US. *International Classification of Diseases 9*^*th*^*/10*^*th*^ *Revision* (ICD-9/10) diagnosis codes (427.3, I48) were used to identify all adult patients (≥18 years) with AF. Subsequently, patients who underwent isolated mitral, aortic, tricuspid, and pulmonary valve replacement or repair or multivalve operations were identified using ICD-9/10 procedure codes as previously reported (S1 Table) [13, 14]. The study cohorts were then stratified based on the presence or absence of ICD-9/10 procedure codes for LAAO (*LAAO*, *nLAAO)* corresponding to concomitant LAAO utilization. To maintain homogeneity, patients undergoing heart transplantation, durable ventricular assist device placement, transcatheter valve operations or with history of endocarditis were excluded.

Patient and hospital characteristics including age, sex, race, income quartile, primary payer, hospital region and teaching status were defined in accordance with the HCUP data dictionary [12]. Comorbidities including congestive heart failure, coronary artery disease, peripheral vascular disease, pulmonary circulation disease, chronic lung disease, diabetes, hypertension, hypothyroidism, and chronic kidney disease, as well as chronic anticoagulation use were identified using ICD-9/10 diagnosis codes. Additionally, the cumulative burden of chronic conditions was quantified using the Van Walraven modification of the Elixhauser Comorbidity Index, a validated composite of 30 comorbidities [15, 16]. A CHA_2_DS_2_VASc score was computed for each patient as a validated estimation of the yearly risk of stroke in patients with AF (S1 Table) [17]. ICD-9/10 procedure codes were used to ascertain robot-assisted operations and concomitant maze procedures (S1 Table). Perioperative complications included cerebrovascular (stroke), thromboembolic (deep vein thrombosis, pulmonary embolism), infectious (sepsis), pulmonary (respiratory failure, prolonged mechanical ventilation), cardiac (cardiac arrest, cardiogenic shock), and renal (acute kidney injury). Hospitalization costs were generated by applying center-specific cost-to-charge ratios to overall charges and were inflation-adjusted to the 2019 Personal Health Care Index [18]. The primary outcome of this study was LAAO utilization, while in-hospital mortality, complications, hospitalization costs and length of stay (LOS) were also assessed.

Categorical variables are reported as frequencies (%) while continuous variables are summarized as medians with interquartile range [IQR]. To assess significance of differences across groups, we used the Pearson’s chi-squared test for categorical variables and the adjusted Wald and Mann-Whitney U test for continuous ones. Significance of temporal trends was assessed using Cuzick’s nonparametric test (nptrend) [19]. Multivariable linear and logistic regression models were developed to evaluate the association of LAAO utilization with patient and hospital characteristics, mortality, complications, costs and LOS. Variable selection was guided by application of the Least Absolute Shrinkage and Selection Operator (LASSO) to reduce collinearity among covariates while decreasing overfitting [20]. Optimization of the final model was based on minimization of the root mean squared error term on 10-fold cross validation as well as maximization of the area under the receiver-operating characteristics curve (C-statistic), as appropriate. Regression outcomes are reported as adjusted odds ratios (AOR) for categorical variables or beta coefficients (β) for continuous variables with 95% confidence intervals (95% CI). Predicted estimates were generated using the Stata margins command after regression.

Statistical significance was set at α = 0.05. All statistical analyses were performed using Stata 16.1 (StataCorp, College Station, TX). Due to the de-identified nature of the NIS, this study did not require informed consent and was deemed exempt from full review by the Institutional Review Board at the University of California, Los Angeles.

## Results

Of an estimated 382,580 patients with AF undergoing valve operations, 71,510 (18.7%) underwent concomitant LAAO. Among the *LAAO* cohort, the most common concurrent operations were mitral (65.8%) and aortic (42.5%) valve operations, while 32.3% of the *LAAO* cohort also had a maze procedure (Table 1). Compared to *nLAAO*, *LAAO* patients had similar age (median: 71 years), burden of comorbidities, and racial demographics (Table 1). Additionally, the *LAAO* cohort had a greater proportion of female patients relative to *nLAAO* (41.6 vs 37.3%, p<0.001). *LAAO* patients more frequently had chronic anticoagulation use relative to *nLAAO* (30.8 vs 17.4%, p<0.001), and both cohorts had similar CHA_2_DS_2_VASc scores (median: 4) indicating high risk of stroke. Furthermore, a lower proportion of *LAAO* operations were performed in the South (31.1 vs 34.2%) and Northeast (17.6 vs 23.0%), while the Midwest (28.1 vs 22.7%) and West (23.2 vs 20.1%) had a greater proportion compared to *nLAAO* (p<0.001). There was no significant difference in LAAO utilization rates between rural and metropolitan hospitals (Table 1).

**Table 1:** Patient, operative and hospital characteristics stratified by utilization of left atrial appendage occlusion (LAAO) during valvular heart surgery. *IQR*: *Interquartile range*. *LAAO*: *concomitant LAAO use*. *nLAAO*: *no concomitant LAAO use*.

Parameter	nLAAO (n = 311,070)	LAAO (n = 71,510)	*p*-value
Age (years, median, IQR)	71 [63–77]	71 [63–77]	0.24
Female sex (%)	37.3	41.6	<0.001
*Race (%)*			<0.001
White	83.4	83.1	
Black	5.5	5.3	
Hispanic	5.5	5.4	
Asian	2.4	3.4	
Other	3.2	2.8	
*Comorbidities (%)*			
Elixhauser Comorbidity Index (median, IQR)	5 [4–6]	5 [4–7]	<0.001
Congestive heart failure	49.5	57.9	<0.001
Coronary artery disease	55.2	50.4	<0.001
Peripheral vascular disorder	23.1	15.0	<0.001
Pulmonary circulation disorder	20.9	29.2	<0.001
Chronic lung disease	29.4	32.6	<0.001
Diabetes	27.7	25.1	<0.001
Hypertension	77.9	75.6	<0.001
Hypothyroidism	13.7	14.9	<0.001
Chronic kidney disease	2.6	2.0	<0.001
Chronic anticoagulation use	17.4	30.8	<0.001
CHA_2_DS_2_VASc Score (median, IQR)	4 [3–5]	4 [3–5]	0.15
*Income Quartile (%)*			0.01
Fourth (highest)	26.1	26.9	
Third	26.0	26.2	
Second	25.5	25.9	
First (lowest)	22.4	21.0	
*Payer Status (%)*			0.17
Private	24.7	24.5	
Medicare	67.1	67.6	
Medicaid	4.7	4.8	
Other	3.5	3.1	
Robot-assisted (%)	0.40	0.43	0.71
*Concomitant Operation (%)*			
Mitral valve	37.9	65.8	<0.001
Aortic valve	67.5	42.5	<0.001
Tricuspid valve	7.6	14.1	<0.001
Pulmonic valve	0.34	0.16	<0.001
Multi-valve	12.3	20.8	<0.001
Maze	12.5	32.3	<0.001
*Hospital Region (%)*			<0.001
South	34.2	31.1	
Northeast	23.0	17.6	
Midwest	22.7	28.1	
West	20.1	23.2	
*Hospital Teaching Status (%)*			0.05
Non-metropolitan	1.8	1.6	
Metropolitan non-teaching	16.1	17.3	
Metropolitan teaching	82.0	81.1	

**Fig 1:**
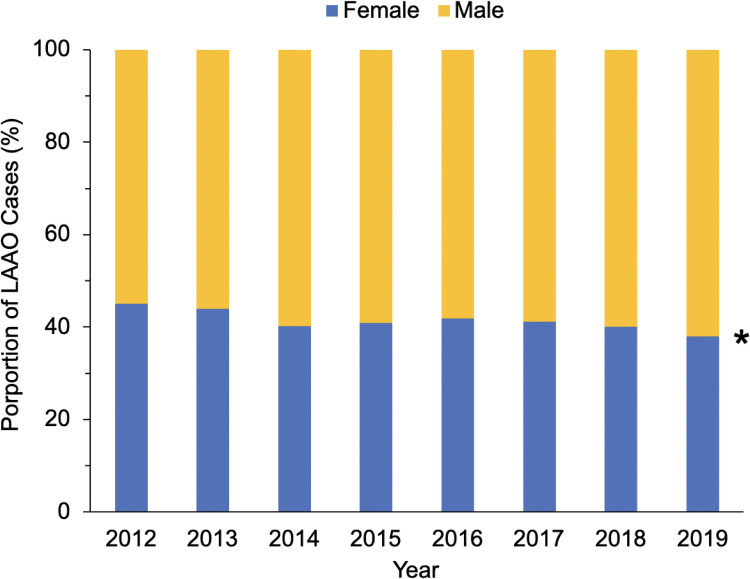
Temporal trends in the proportion of left atrial appendage occlusion (LAAO) cases stratified by sex. *Nptrend = 0.*001*.

Over the study period, the overall utilization rate of LAAO remained steady (S1 Fig). On subgroup analysis, the proportion of female patients who underwent LAAO significantly decreased from 44.8% to 38.9% over the 8-year study period (nptrend = 0.001, Fig 1). The proportion of LAAO cases by race and hospital region had no significant temporal trends.

**Fig 2:**
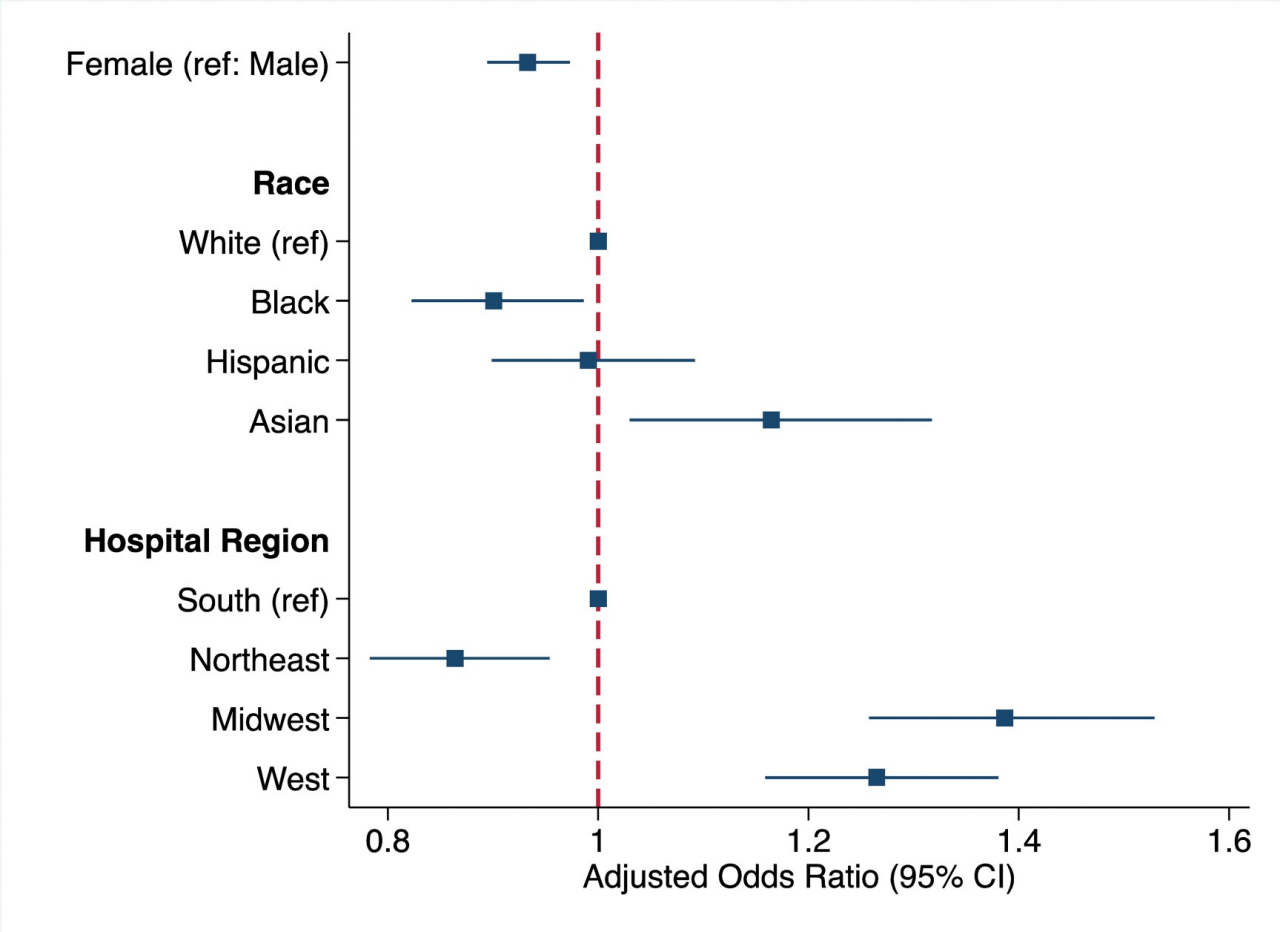
Patient and hospital characteristics associated with utilization of left atrial appendage occlusion during valvular heart surgery. Error bars represent 95% confidence intervals. Model C-statistic: 0.72. *Ref*: *Reference*. *CI*: *Confidence interval*.

On multivariable logistic regression (C-statistic = 0.72), females had 7% lower likelihood of undergoing LAAO compared to males (AOR 0.93, 95% CI 0.89–0.97). Relative to Whites, Black patients had significantly reduced odds of LAAO (AOR 0.91, 95% CI 0.83–0.99), while Hispanic and Asian patients had comparable (AOR 0.99, 95% CI 0.90–1.09) and increased odds (AOR 1.16, 95% CI 1.03–1.32) of undergoing LAAO, respectively (Fig 2). In addition, hospitals in the Midwest (AOR 1.38, 95% CI 1.24–1.51) and West (AOR 1.26, 95% CI 1.15–1.36) had significantly increased likelihood of LAAO whereas Northeast hospitals (AOR 0.85, 95% CI 0.77–0.94) had decreased odds relative to the South. Additional patient, operative, and hospital characteristics associated with utilization of LAAO during valvular heart surgery are tabulated in S2 Table, including operative type. Primary payer, hospital teaching status, and hospital volume of valve operations had no significant association with LAAO use.

Unadjusted clinical and financial outcomes are shown in Table 2. Compared to *nLAAO*, *LAAO* individuals experienced lower in-hospital mortality rates (2.9 vs 3.4%, p = 0.002). In addition, the *LAAO* cohort had significantly reduced rates of stroke (1.2 vs 2.0%, p<0.001) and thromboembolic (0.61 vs 0.92%, p<0.001) complications. Septic (3.3 vs 3.8%, p = 0.001) and respiratory (19.1 vs 20.1%, p = 0.01) complication rates were also significantly decreased in the *LAAO* group. However, the incidence of cardiac arrest or shock as well as acute kidney injury remained comparable across the cohorts. Additionally, unadjusted LOS and hospitalization costs were similar in number between the *nLAAO* and *LAAO* groups (Table 2).

**Table 2:** Unadjusted in-hospital clinical and financial outcomes stratified by utilization of left atrial appendage occlusion (LAAO) during valvular heart surgery. *IQR*: *Interquartile range*. *LAAO*: *concomitant LAAO use*. *nLAAO*: *no concomitant LAAO use*.

Outcome	nLAAO (n = 311,070)	LAAO (n = 71,510)	p-value
In-hospital mortality (%)	3.4	2.9	0.002
*Complications (%)*			
Stroke	2.0	1.2	<0.001
Thromboembolic	0.92	0.61	<0.001
Sepsis	3.8	3.3	0.001
Respiratory	20.1	19.1	0.01
Cardiac arrest / shock	8.5	8.4	0.74
Acute kidney injury	23.1	22.8	0.48
Length of stay (days, median, IQR)	8 [6–13]	8 [6–13]	0.001
Cost ($1000s, median, IQR)	51.6 [38.8–72.9]	52.6 [39.9–72.0]	<0.001

**Fig 3:**
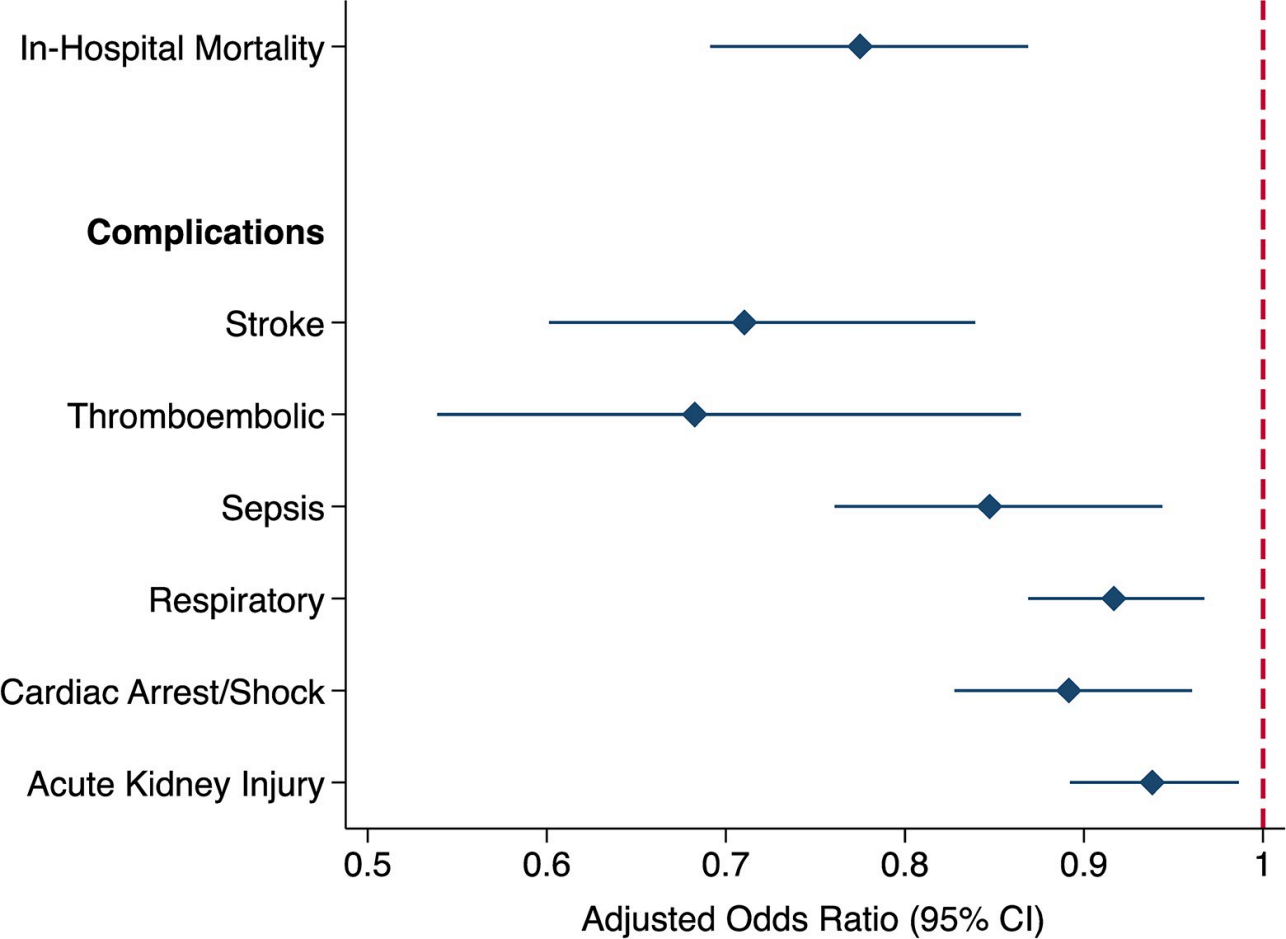
Adjusted in-hospital clinical outcomes associated with left atrial appendage occlusion utilization during valvular heart surgery. *CI*: *Confidence interval*.

**Fig 4:**
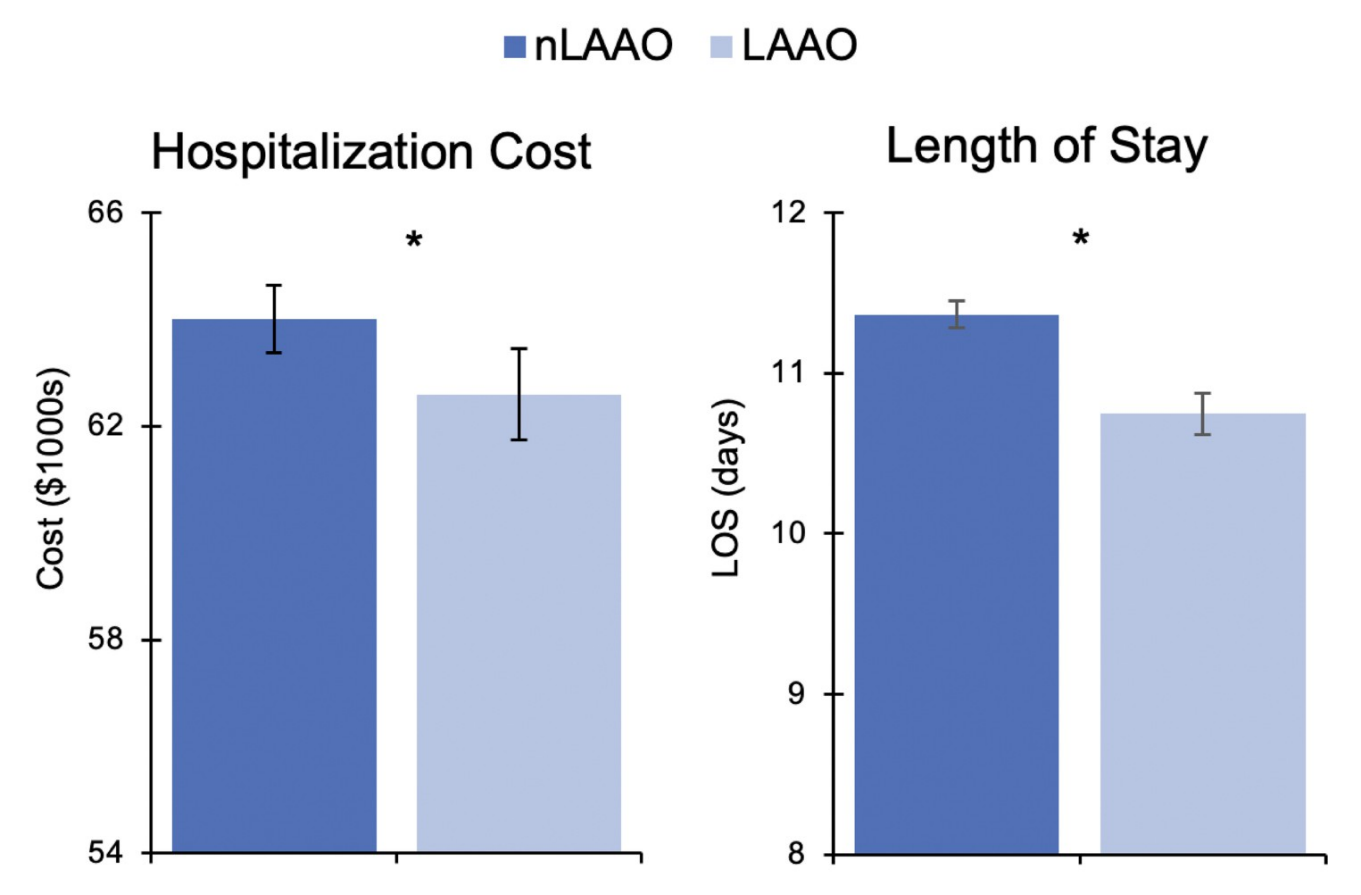
Risk-adjusted hospitalization costs and length of stay (LOS) associated with left atrial appendage occlusion (LAAO) utilization during valvular heart surgery. *p<0.*001*. *LAAO*: *concomitant LAAO use*. *nLAAO*: *no concomitant LAAO use*.

Following adjustment for risk factors listed in S2 Table, concomitant LAAO utilization was associated with 22% decreased odds of in-hospital mortality (AOR 0.78, 95% CI 0.69–0.87, Fig 3). Furthermore, patients with LAAO had significantly reduced odds of stroke (AOR 0.71, 95% CI 0.60–0.84) and thromboembolism (AOR 0.68, 95% CI 0.54–0.86). *LAAO* was associated with significantly lower likelihood of all other perioperative complications including sepsis (AOR 0.85, 95% CI 0.76–0.94), respiratory complications (AOR 0.92, 95% CI 0.87–0.97), cardiac arrest or shock (AOR 0.89, 95% CI 0.83–0.96), and acute kidney injury (AOR 0.94, 95% CI 0.89–0.99, Fig 3). In addition, *LAAO* patients had significantly reduced attributable hospitalization costs ($62,600 [95% CI 61,700–63,400] vs $64,000 [63,400–64,700], p<0.001) and a 1-day decrement in LOS (10.7 days [10.6–10.9] vs 11.4 [11.3–11.4], p<0.001, Fig 4).

## Discussion

Using a nationally representative cohort of patients with AF undergoing heart valve operations, we examined the association of concomitant LAAO utilization with sex, race, hospital region, as well as in-hospital clinical and financial outcomes. Consistent with prior literature, the most common valvular operations among the LAAO cohort were mitral (65.8%) and aortic (42.5%) valve operations, while 32.3% also had a maze procedure [21, 22]. Over the study period, the proportion of female patients undergoing LAAO during valve operations significantly decreased. Upon risk adjustment, female and Black patients had lower likelihood of receiving LAAO compared to males and Whites, respectively. Additionally, patients at hospitals in the Midwest and West had greater odds whereas the Northeast had lower odds of LAAO use relative to the South. Overall, patients with LAAO experienced reduced in-hospital mortality and stroke as well as decreased costs and LOS. Several of these findings warrant further discussion.

The present study revealed that female patients had 7% lower likelihood of receiving LAAO compared to males. Although mitral valve procedures are predominantly performed in women [23] and females had higher unadjusted rates of LAAO use, controlling for patient and operative factors revealed significant sex-based disparities. Prior studies have reported underutilization of cardiac operations such as surgical aortic valve replacement among women [24]. Moreover, the proportion of women receiving LAAO steadily decreased from 44.8% to 38.9% over the 8-year study period. Growing evidence suggests that females are at greater risk of adverse events such as pericardial effusion and major bleeding following LAAO, which may discourage offering the procedure [9, 25, 26]. Anatomical differences, increased frailty, as well as physician inexperience, particularly with women who may be at inherently increased risk, have been cited as factors contributing to such sex-based differences [9]. The persistent disparity in treatment over time underscores the need for further clinical trial data of LAAO use during valve operations to adequately guide sex-based safety guidelines and effective outcomes.

Significant racial bias in LAAO utilization was also observed. Relative to Whites, Black patients had 9% reduced odds of undergoing LAAO during valvular surgery. Our findings add to a body of literature on racial disparities in the utilization of cardiac procedures, including independent valve replacement and transcatheter LAAO [8, 27]. The reasons are likely multifactorial. Black patients are less likely to follow guideline-directed anticoagulation therapy for AF management, and those with severe aortic stenosis often decline aortic valve replacement against recommendation, suggesting issues concerning historical discrimination, trust, and systemic racism in the delivery of care [28, 29]. Lower socioeconomic status often underlies disparate access to care among racial and ethnic minorities, but the present work found no significant association between LAAO utilization and payer [29]. Additional efforts to mitigate racial bias in AF treatment and identify causal factors at the patient, provider, and institutional levels are warranted.

Hospitals in the Midwest and West had significantly increased likelihood of LAAO utilization whereas Northeast hospitals had decreased odds compared to the South. These findings align with Friedman et al.’s previous analysis of a smaller study cohort, which found the highest utilization rates of LAAO during cardiac surgery in the Great Lakes (25%) and lowest in New England (5%) [30]. Interestingly, we found that hospital metropolitan teaching status as well as operative volume had no significant impact on LAAO utilization, indicating that these geographic disparities may be due to other population-based characteristics. Southern states have the highest proportion of Black individuals, which may explain the reduced use of LAAO [31]. In addition, prior studies that have similarly found Western states more likely to receive cardiac operations have cited state certificate-of-need (CON) policies, which still regulate provision of cardiac procedures in many states except those in the West [32].

Notably, we found that utilization of LAAO during valvular operations was associated with over 20% decreased odds of in-hospital mortality, stroke, and thromboembolism. Opinions regarding the efficacy of LAAO have been mixed largely due to the technical challenge of achieving complete appendage closure [33]. However, increasing evidence from randomized controlled trials in recent years have consistently found lower risk of ischemic stroke and systemic embolism among patients with AF undergoing concomitant LAAO and cardiac procedures [11, 21, 34]. Literature suggests that LAAO may improve fibrin clot permeability and susceptibility to lysis in the cardiovascular system [35]. Additionally, LAAO has been associated with neurohormonal changes in the renin-angiotensin-aldosterone system favoring blood pressure reduction [36], potentially underlying the improvement in renal, cardiac, respiratory, and septic complications observed in our study. Moreover, we found significantly reduced hospitalization costs ($62,600 vs $64,000) and a 1-day decrement in LOS among LAAO patients, exemplifying improved financial outcomes. As the potential benefits of undergoing LAAO during valvular surgery come to light, it is imperative to better understand sociodemographic disparities in utilization and their influence on patient outcomes.

The present study has several limitations inherent to its retrospective nature and use of administrative data. The NIS lacks clinical granularity regarding information such as symptom severity, intraoperative techniques and true completion of appendage occlusion. Notably, the type, dosage, duration, and adherence to anticoagulation therapy were unable to be captured in the NIS database. Variation in coding practices at participating hospitals could have also contributed to misclassification bias or incomplete data. Furthermore, ICD-9/10 codes for AF (427.3, I48) were unable to stratified into different types and included paroxysmal, chronic, preoperative and postoperative AF. This likely contributed to underestimation of the true rates of chronic anticoagulation use. Nevertheless, we defined AF using methodology that has been validated and reviewed in prior studies using administrative data [37, 38]. Similarly, excision, destruction, and occlusion of LAA were defined by a singular ICD-9 procedure code (37.36) and could not be stratified. All available data were limited to the duration of hospitalization and did not capture long-term outcomes, which may have underestimated the true incidence of complications and mortality as well as need for readmission. Potential factors that may influence surgeons’ decisions to perform concomitant LAAO were not available, including history of bleeding disorders or occurrence of intraoperative complications. Despite these limitations, we utilized the largest all-payer inpatient database and robust statistical methods to enhance the generalizability of our findings at the national level.

In conclusion, the present study used a nationally representative database to demonstrate that female and Black patients had significantly lower odds of LAAO utilization, while hospitals in the Midwest and West were associated with greater use of LAAO during valvular heart surgery. Given the improved in-hospital clinical and financial outcomes associated with LAAO, increasing access to LAAO should be considered for patients with AF across the United States.

## Supporting information

S1 Table Administrative *International Classification of Diseases*, *9*^*th*^ *and 10*^*th*^ *Revision* (ICD-9/10) diagnosis and procedure codes for cardiac operations, baseline patient characteristics, and in-hospital outcomes. (DOCX)

S2 Table Patient, operative and hospital characteristics associated with utilization of left atrial appendage occlusion during valvular heart surgery. Model C-statistic: 0.72. *Ref*: *Reference*. *AOR*: *Adjusted odds ratio*. *CI*: *Confidence interval*.
(DOCX)

S1 Fig Temporal trends in the rate of concomitant left atrial appendage occlusion (LAAO) utilization in patients with AF undergoing heart valve operations. Nptrend = 0.15. *LAAO*: *concomitant LAAO use*. *nLAAO*: *no concomitant LAAO use*.
(DOCX)

## References

[1] Chugh SS, Havmoeller R, Narayanan K, et al. Worldwide epidemiology of atrial fibrillation: a Global Burden of Disease 2010 Study. *Circulation*. 2014;129(8):837–847. doi: 10.1161/CIRCULATIONAHA.113.005119 24345399 PMC4151302

[2] Ball J, Carrington MJ, McMurray JJ, Stewart S. Atrial fibrillation: profile and burden of an evolving epidemic in the 21st century. *Int J Cardiol*. 2013;167(5):1807–1824. doi: 10.1016/j.ijcard.2012.12.093 23380698

[3] Hart RG, Pearce LA, Aguilar MI. Meta-analysis: antithrombotic therapy to prevent stroke in patients who have nonvalvular atrial fibrillation. *Ann Intern Med*. 2007;146(12):857–867. doi: 10.7326/0003-4819-146-12-200706190-00007 17577005

[4] Hsu JC, Maddox TM, Kennedy KF, et al. Oral anticoagulant therapy prescription in patients with atrial fibrillation across the spectrum of stroke risk: insights from the NCDR PINNACLE Registry. *JAMA Cardiol*. 2016;1(1):55–62. doi: 10.1001/jamacardio.2015.0374 27437655

[5] Fuster V, Ryden LE, Cannom DS, et al. 2011 ACCF/AHA/HRS focused updates incorporated into the ACC/AHA/ESC 2006 Guidelines for the management of patients with atrial fibrillation: a report of the American College of Cardiology Foundation/American Heart Association task force on practice guidelines developed in partnership with the European Society of Cardiology and in collaboration with the European Heart Rhythm Association and the Heart Rhythm Society. *J Am Coll Cardiol*. 2011;57:e101–98. doi: 10.1016/j.jacc.2010.09.013 21392637

[6] January CT, Wann LS, Alpert JS, et al; American College of Cardiology/American Heart Association Task Force on Practice Guidelines. 2014 AHA/ACC/HRS guideline for the management of patients with atrial fibrillation: a report of the American College of Cardiology/American Heart Association Task Force on Practice Guidelines and the Heart Rhythm Society. *J AmColl Cardiol*. 2014;64(21):e1–e76. 10.1016/j.jacc.2014.03.022 24685669

[7] Kirchhof P, Benussi S, Kotecha D, et al. 2016 ESC Guidelines for the management of atrial fibrillation developed in collaboration with EACTS. *Eur Heart J*. 2016;37(38):2893–2962. doi: 10.1093/eurheartj/ehw210 27567408

[8] Sparrow R, Sanjoy S, Choi YH, et al. Racial, ethnic and socioeconomic disparities in patients undergoing left atrial appendage closure. *Heart*. 2021;107(24):1946–55. doi: 10.1136/heartjnl-2020-318650 33795381

[9] Darden D, Duong T, Du C, et al. Sex differences in procedural outcomes among patients undergoing left atrial appendage occlusion: insights from the NCDR LAAO registry. *JAMA Cardiology*. 2021;6(11):1275–84. doi: 10.1001/jamacardio.2021.3021 34379072 PMC8358791

[10] Lanska DJ, Kuller LH. The geography of stroke mortality in the United States and the concept of a stroke belt. *Stroke*. 1995;26:1145–1149. doi: 10.1161/01.str.26.7.1145 7604404

[11] Whitlock RP, Belley-Cote EP, Paparella D, et al. Left atrial appendage occlusion during cardiac surgery to prevent stroke. *New England Journal of Medicine*. 2021;384(22):2081–91. doi: 10.1056/NEJMoa2101897 33999547

[12] HCUP National Inpatient Sample (NIS). Healthcare Cost and Utilization Project (HCUP), Agency for Healthcare Research and Quality, Rockville, MD. 2012 (accessed 23 Dec 2022). Retrieved from www.hcup-us.ahrq.gov/nisoverview.jsp.

[13] Hadaya J, Sanaiha Y, Hernandez R, Tran Z, Shemin RJ, Benharash P. Impact of hospital volume on resource use after elective cardiac surgery: A contemporary analysis. *Surgery*. 2021;170(3):682–8. doi: 10.1016/j.surg.2021.03.004 33849734

[14] Zhou S, Egorova N, Moskowitz G, Giustino G, et al. Trends in MitraClip, mitral valve repair, and mitral valve replacement from 2000 to 2016. *The Journal of Thoracic and Cardiovascular Surgery*. 2021;162(2):551–62. doi: 10.1016/j.jtcvs.2019.12.097 32089343 PMC7957952

[15] Elixhauser A, Steiner C, Harris DR, Coffey RM. Comorbidity measures for use with administrative data. Med Care. 1998;36:8–27, doi: 10.1097/00005650-199801000-00004 9431328

[16] Stagg V. ELIXHAUSER: Stata Module to Calculate Elixhauser Index of Comorbidity. Statistical Software Components S458077, Boston College Department of Economics. 2015. Available online: https://ideas.repec.org/c/boc/bocode/s458077.html (accessed April 15 2023).

[17] Borovac JA, Kwok CS, Mohamed MO, et al. The predictive value of CHA2DS2-VASc score on in-hospital death and adverse periprocedural events among patients with the acute coronary syndrome and atrial fibrillation who undergo percutaneous coronary intervention: a 10-year National Inpatient Sample (NIS) analysis. *Cardiovascular Revascularization Medicine*. 2021;29:61–8. doi: 10.1016/j.carrev.2020.08.003 32828675

[18] Medical Expenditure Panel Survey. Using appropriate price indices for expenditure comparisons. *Agency for Healthcare Research and Quality*. 2021 (accessed 23 Dec 2022). Retrieved from https://meps.ahrq.gov/about_meps/Price_Index.shtml

[19] Cuzick J. A wilcoxon‐type test for trend. *Stat Med*. 1985;4:543–547. doi: 10.1002/sim.4780040416 4089356

[20] Tibshirani R. Regression shrinkage and selection via the lasso. *J R Stat Soc Ser B*. 1996;58:267–288.

[21] Elbadawi A, Olorunfemi O, Ogunbayo GO, et al. Cardiovascular outcomes with surgical left atrial appendage exclusion in patients with atrial fibrillation who underwent valvular heart surgery (from the National Inpatient Sample Database). *The American Journal of Cardiology*. 2017;119(12):2056–60. doi: 10.1016/j.amjcard.2017.03.037 28438308

[22] Melduni RM, Schaff HV, Lee HC, et al. Impact of left atrial appendage closure during cardiac surgery on the occurrence of early postoperative atrial fibrillation, stroke, and mortality: a propensity score–matched analysis of 10 633 patients. *Circulation*. 2017;135(4):366–78. 27903589 10.1161/CIRCULATIONAHA.116.021952 PMC5469206

[23] Redberg RF, Schiller NB. Gender and valvular surgery (editorial). *Journal of Thoracic and Cardiovascular Surgery*. 2004;127(1): 1–3. 14752403 10.1016/j.jtcvs.2003.09.022

[24] Chaker Z, Badhwar V, Alqahtani F, et al. Sex differences in the utilization and outcomes of surgical aortic valve replacement for severe aortic stenosis. *Journal of the American Heart Association*. 2017;6(9):e006370. doi: 10.1161/JAHA.117.006370 28935681 PMC5634288

[25] Abusnina W, Latif A, Al-Abdouh A, et al. Sex differences in the clinical outcomes after left atrial appendage closure: A systematic review and meta-analysis. *Cardiovascular Revascularization Medicine*. 2022;41:29–34. doi: 10.1016/j.carrev.2021.12.013 34952822

[26] Sanjoy SS, Choi YH, Sparrow RT, et al. Sex differences in outcomes following left atrial appendage closure. *Mayo Clinic Proceedings*. 2021;96(7):1845–1860. doi: 10.1016/j.mayocp.2020.11.031 34218859

[27] Vincent L, Grant J, Ebner B, et al. Racial disparities in the utilization and in-hospital outcomes of percutaneous left atrial appendage closure among patients with atrial fibrillation. *Heart rhythm*. 2021;18(6):987–94. doi: 10.1016/j.hrthm.2021.02.008 33588068

[28] Alkhouli M, Holmes DR, Carroll JD, et al. Racial disparities in the utilization and outcomes of TAVR: TVT Registry report. *JACC Cardiovasc Interv*. 2019;12:936–48. doi: 10.1016/j.jcin.2019.03.007 31122351

[29] Sleder A, Tackett S, Cerasale M, et al. Socioeconomic and racial disparities: a case-control study of patients receiving transcatheter aortic valve replacement for severe aortic stenosis. *J Racial Ethn Health Disparities*. 2017;4:1189–94. doi: 10.1007/s40615-016-0325-x 28039604

[30] Friedman DJ, Piccini JP, Wang T, et al. Association between left atrial appendage occlusion and readmission for thromboembolism among patients with atrial fibrillation undergoing concomitant cardiac surgery. *JAMA*. 2018;319(4):365–74. doi: 10.1001/jama.2017.20125 29362794 PMC5833567

[31] S Rastogi TD Johnson, Hoeffel EM, Drewery MP Jr. The Black Population: 2010. U.S. Census Bureau 2010 Census interactive population search. 2011 (accessed Dec 23, 2022). Retrieved from www.census.gov/prod/cen2010/briefs/c2010br-06.pdf

[32] Trivedi AN, Sequist TD, Ayanian JZ. Impact of hospital volume on racial disparities in cardiovascular procedure mortality. *JACC*. 2006;47(2):417–24. doi: 10.1016/j.jacc.2005.08.068 16412871

[33] Healey JS, Crystal E, Lamy A, et al. Left Atrial Appendage Occlusion Study (LAAOS): results of a randomized controlled pilot study of left atrial appendage occlusion during coronary bypass surgery in patients at risk for stroke. *Am Heart J*. 2005;150:288–93. doi: 10.1016/j.ahj.2004.09.054 16086933

[34] Prasad RM, Saleh Y, Al-Abcha A, et al. Left atrial appendage closure during cardiac surgery for atrial fibrillation: A meta-analysis. *Cardiovascular Revascularization Medicine*. 2022;40:26–36. doi: 10.1016/j.carrev.2021.11.002 34801420

[35] Bartus K, Kanuri SH, Litwinowicz R, et al. Long term impact of epicardial left atrial appendage ligation on systemic hemostasis: LAA HOMEOSTASIS-2. *J Clin Med*. 2022;11(6):1495. doi: 10.3390/jcm11061495 35329819 PMC8955343

[36] Litwinowicz R, Natorska J, Zabczyk M, et al. Changes in Fibrinolytic Activity and Coagulation Factors after Epicardial Left Atrial Appendage Closure in Patients With Atrial Fibrillation. *J Thorac Dis*. 2022;14(11):4226–4235. 36524072 10.21037/jtd-21-1093 PMC9745526

[37] Jensen PN, Johnson K, Floyd J, Heckbert SR, Carnahan R, Dublin S. A systematic review of validated methods for identifying atrial fibrillation using administrative data. *Pharmacoepidemiology and Drug Safety*. 2012;21:141–7. doi: 10.1002/pds.2317 22262600 PMC3674852

[38] McCarthy PM, Davidson CJ, Kruse J, et al. Prevalence of atrial fibrillation before cardiac surgery and factors associated with concomitant ablation. *Journal of Thoracic and Cardiovascular Surgery*. 2020;159(6):2245–53. doi: 10.1016/j.jtcvs.2019.06.062 31444073

