# Supplementary material for: Sociodemographic disparities in concomitant left atrial appendage occlusion during cardiac valve operations
Source: PLoS One. 2023 May 25;18(5):e0286337. doi: 10.1371/journal.pone.0286337 (PMC10212171; doi:10.1371/journal.pone.0286337)
Supplement: S1 Table — (DOCX) [file pone.0286337.s001.docx]

Supplemental Table 1. Administrative *International Classification of Diseases, 9^th^ and 10^th^ Revision* (ICD-9/10) diagnosis and procedure codes for cardiac operations, baseline patient characteristics, and in-hospital outcomes.

|  | **ICD-9** | **ICD-10** |
| --- | --- | --- |
| **Cardiac Operations** |  |  |
| Aortic Valve Replacement | 35.21, 35.22 | 02RF07x, 02RF08x, 02RF0Jx, 02RF0Kx, 02RF47x, 02RF48x, 02RF4Jx, 02RF4Kx, |
| Aortic Valve Repair | 35.11 | 02QF0Zx 02QF4Zx, 02UF07x, 02UF08x, 02UF0Jx, 02UF0K, 02UF47x, 02UF48x, 02UF4Jx, 02UF4Kx |
| Mitral Valve Replacement | 35.23, 35.24 | 02RG07x, 02RG08x, 02RG0Jx, 02RG0Kx  02RG47x, 02RG48x, 02RG4Jx, 02RG4Kx |
| Mitral Valve Repair | 35.12 | 02QG0Z, 02QG4Z,  02UG07x, 02UG08x, 02UG0Jx, 02UG0Kx  02UG47x, 02UG48x, 02UG4Jx, 02UG4Kx |
| Tricuspid Valve Replacement | 35.27, 35.28 | 02RJ07x, 02RJ08x, 02RJ0Jx, 02RJ0Kx  02RJ47x, 02RJ48x, 02RJ4Jx, 02RJ4Kx |
| Tricuspid Valve Repair | 35.14 | 02QJ0Z, 02QJ4Z |
| Pulmonary Valve Replacement | 35.25, 35.26 | 02UJ07x, 02UJ08x, 02UJ0Jx, 02UJ0Kx  02UJ47x, 02UJ48x, 02UJ4Jx, 02UJ4Kx |
| Pulmonary Valve Repair | 35.13 | 02QH0x, 02QH4Zx,  02UH07x, 02UH08x, 02UH0Jx, 02UH0Kx  02UH47x, 02UH48x, 02UH4Jx, 02UH4Kx |
| Transcatheter Mitral Valve Interventions | 35.97 | 02UG3JZ, 02QG3ZZ, 02RG3JZ, 02UG37E, 02UG37Z, 02UG38E, 02UG38Z, 02UG3JE, 02UG3JZ, 02UG3KE, 02UG3KZ, 02RG37H, 02RG37Z, 02RG38H, 02RG38Z, 02RG3JH, 02RG3JZ, 02RG3KH, 02RG3KZ |
| Transcatheter Aortic Valve Replacement or Repair | 35.05, 35.06 | 02RF37H, 02RF37Z, 02RF38H, 02RF38Z, 02RF3JH, 02RF3JZ, 02RF3KH, 02RF3KZ |
| Transcatheter Pulmonary Valve Replacement or Repair | 35.07, 35.08 | 02RH37H, 02RH37Z, 02RH38H, 02RH38Z, 02RH3JH, 02RH3JZ, 02RH3KH, 02RH3KZ |
| Transcatheter Tricuspid Valve Replacement or Repair | -- | 02RJ37H, 02RJ37Z, 02RJ38H, 02RJ38Z, 02RJ3JH, 02RJ3JZ, 02RJ3KH, 02RJ3KZ |
| Other Transcatheter Valve Procedure | 35.09 | -- |
| Left Atrial Appendage Occlusion | 37.36 | 02570ZK, 02B70ZK, 02L70ZK |
| Maze Procedure | 37.33, 37.34, 37.37 | 02560ZZ, 02563ZZ, 02564ZZ, 02570ZZ, 02573ZZ, 02574ZZ, 02580ZZ, 02583ZZ, 02584ZZ, 02B60ZZ, 02B63ZZ, 02B64ZZ, 02B70ZZ, 02B73ZZ, 02B74ZZ, 02B80ZZ, 02B83ZZ, 02B84ZZ |
| Robot-Assisted | 17.42, 17.44 | 8E0W4CZ, 8E0W8CZ |
| Heart Transplant | 37.51 | 02YA0Z0 |
| Durable Ventricular Assist Device Placement | 37.66, 37.63 | 02HA0QZ, 02WA0QZ, 02WA3QZ, 02WA4QZ |
|  |  |  |
| **Comorbidities** |  |  |
| Endocarditis | 42.1, 42.49 | I33, I38, I39 |
| Congestive Heart Failure | 398.91, 402.01, 402.91, 404.01, 404.03, 404.11, 404.13, 404.91, 404.93, 425.4, 425.5, 425.7, 425.8, 425.9, 428 | I43, I50, I09.9, I11.0, I13.0, I25.5, I42.0, I42.5, I42.6, I42.7, I42.8, I42.9, P29.0 |
| Coronary Artery Disease | 411, 412, 413, 414 | I20, I25 |
| Peripheral Vascular Disease | 093.0, 440, 441, 437.3, 443, 447.1, 557.1, 557.9 | I70, I71, I73, I77 |
| Pulmonary Circulation Disease | 416, 415.0, 415.1, 417.0, 417.8, 417.9 | I26, I27, I28 |
| Chronic Lung Disease | 416.8, 416.9, 506.4, 508.8, 500, 501, 502, 503, 504, 505, 490, 491, 492, 493, 494, 495, 496 | I27.8, I27.9, J68.4, J70.1, J70.3, J40, J41, J42, J43, J44, J45, J46, J47, J60, J61, J62, J63, J64, J65, J66, J67 |
| Diabetes | 250 | E10, E11, E13 |
| Hypertension | 401, 402, 403, 404, 405 | I10, I11, I12, I13, I15 |
| Hypothyroidism | 24.09, 24.61, 24.68, 243, 244 | E00, E01, E02, E03, E89.0 |
| Chronic Kidney Disease | 403.01, 403.11, 403.91, 404.02, 404.03, 404.12, 404.13, 404.92, 404.93, 588.0, 585.5, 585.6, 586.5, 586.6 | Z49, N19, I12.0, N25.0, Z94.0, N18.5, N18.6, I13.11, V45.1, Z99.2, Z91.15 |
| Chronic Anticoagulation Use | V58.61 | Z79.01 |
| Previous Cerebrovascular Event (TIA or stroke) | V12.54 | Z86.73 |
| Previous Venous Thrombosis and Embolism | V12.51 | Z86.71 |
| Previous Myocardial Infarction | 412 | I25.2 |
|  |  |  |
| **Complications** |  |  |
| Stroke | 433.01, 433.11, 433.21, 433.31, 433.81, 433.91, 434.01, 434.11, 434.91, 437.0, 437.1, 437.4, 437.5, 437.7, 437.9, 997.01, 997.02, 431, 432.0, 432.1, 432.9, 430 | I63, I67.2, I67.81, I67.82, I67.89, I67.7, I67.5, I67.9, G97.81, G97.82, I97.811, I97.821, I61.9, I62.1, I60.9, I62.9, I62.0, I62.1 |
| Deep Vein Thrombosis | 451.1, 451.2, 451.81, 451.9, 453.2, 453.40, 453.41, 453.42, 453.8, 453.9 | I82.220, I82.4, I82.6, I82.A1, I82.B1, I82.C1, I82.290, I82.890, I82.91, I80.9, I80.3 |
| Pulmonary Embolism | 415.1 | I26 |
| Sepsis | 038, 995.91, 995.92, 999.3, 998.51, 998.59 | A40, A41, R65.20, T814XXA, K68.11 |
| Respiratory Failure | 518.81, 518.51, 518.53, 518.84 | J96.00, J96.90, J96.20, J95.821, J95.822 |
| Prolonged Mechanical Ventilation | 96.72 | 5A1955Z |
| Cardiac Arrest | 427.5 | I46.2, I46.8, I46.9 |
| Cardiogenic Shock | 785.51 | R57.0 |
| Acute Kidney Injury | 584 | N17 |
