# Supplementary material for: Sociodemographic disparities in concomitant left atrial appendage occlusion during cardiac valve operations
Source: PLoS One. 2023 May 25;18(5):e0286337. doi: 10.1371/journal.pone.0286337 (PMC10212171; doi:10.1371/journal.pone.0286337)
Supplement: S2 Table — Model C-statistic: 0.72. Ref: Reference. AOR: Adjusted odds ratio. CI: Confidence interval. (DOCX) [file pone.0286337.s002.docx]

Supplemental Table 2. Patient, operative and hospital characteristics associated with utilization of left atrial appendage occlusion during valvular heart surgery. Model C-statistic: 0.72. *Ref: Reference. AOR: Adjusted odds ratio. CI: Confidence interval.*

| **Parameter** | **AOR [95% CI]** | ***p*-value** |
| --- | --- | --- |
| Age (per year) | 1.01 [1.01-1.01] | <0.001 |
| Female sex | 0.93 [0.89-0.97] | <0.001 |
| *Race* |  |  |
| White | Ref |  |
| Black | 0.91 [0.83-0.99] | 0.02 |
| Hispanic | 0.99 [0.90-1.09] | 0.77 |
| Asian | 1.16 [1.03-1.32] | 0.01 |
| Other | 0.87 [0.76-1.00] | 0.06 |
| *Comorbidities* |  |  |
| Elixhauser Comorbidity Index | 1.03 [1.01-1.05] | <0.001 |
| Congestive heart failure | 1.09 [1.04-1.14] | <0.001 |
| Coronary artery disease | 0.89 [0.86-0.93] | <0.001 |
| Pulmonary circulation disorder | 1.11 [1.05-1.16] | <0.001 |
| Peripheral vascular disorder | 0.80 [0.76-0.85] | <0.001 |
| Diabetes | 0.97 [0.93-1.02] | 0.21 |
| Hypertension | 0.99 [0.94-1.04] | 0.65 |
| Chronic kidney disease | 0.78 [0.68-0.90] | <0.001 |
| Chronic anticoagulation use | 1.65 [1.57-1.73] | <0.001 |
| *Payer Status* |  |  |
| Private | Ref |  |
| Medicare | 0.98 [0.92-1.04] | 0.52 |
| Medicaid | 0.92 [0.83-1.01] | 0.09 |
| Other | 0.87 [0.79-0.98] | 0.02 |
| Year of admission | 0.94 [0.93-0.96] | <0.001 |
| *Concomitant Operation* |  |  |
| Mitral valve | 2.03 [1.71-2.42] | <0.001 |
| Aortic valve | 0.76 [0.65-0.90] | <0.001 |
| Tricuspid valve | 1.09 [0.92-1.30] | 0.32 |
| Pulmonic valve | 0.52 [0.32-0.84] | 0.01 |
| Multi-valve | 1.07 [0.89-1.29] | 0.47 |
| Maze | 2.40 [2.25-2.55] | <0.001 |
| *Hospital Valve Operation Volume Status* |  |  |
| Low volume | Ref |  |
| Medium volume | 0.97 [0.91-1.04] | 0.43 |
| High volume | 0.91 [0.82-1.01] | 0.06 |
| *Hospital Region* |  |  |
| South | Ref |  |
| Northeast | 0.85 [0.77-0.94] | 0.002 |
| Midwest | 1.38 [1.24-1.51] | <0.001 |
| West | 1.26 [1.15-1.36] | <0.001 |
| *Hospital Teaching Status* |  |  |
| Non-metropolitan | Ref |  |
| Metropolitan non-teaching | 1.21 [0.97-1.50] | 0.09 |
| Metropolitan teaching | 1.17 [0.95-1.44] | 0.14 |
