# Supplementary material for: Sociodemographic disparities in concomitant left atrial appendage occlusion during cardiac valve operations
Source: PLoS One. 2023 May 25;18(5):e0286337. doi: 10.1371/journal.pone.0286337 (PMC10212171; doi:10.1371/journal.pone.0286337)

Supplemental Figure 1. Temporal trends in the rate of concomitant left atrial appendage occlusion (LAAO) utilization in patients with AF undergoing heart valve operations. Nptrend = 0.15. *LAAO: concomitant LAAO use. nLAAO: no concomitant LAAO use.*


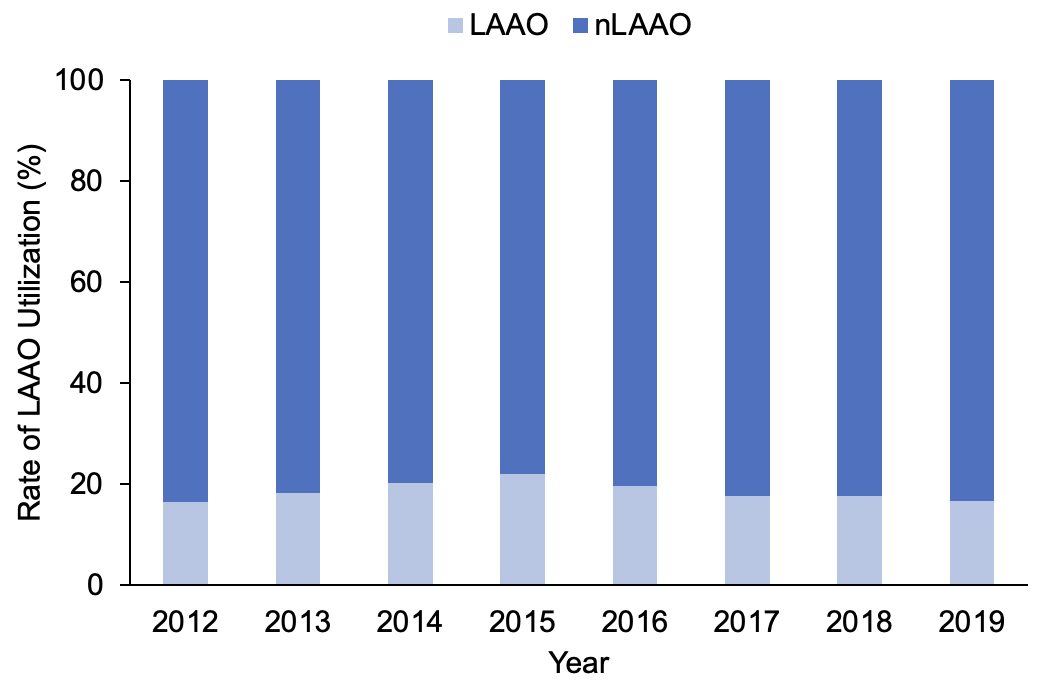

Supplement: S1 Fig — Nptrend = 0.15. LAAO: concomitant LAAO use. nLAAO: no concomitant LAAO use. (DOCX) [file pone.0286337.s003.docx]
